# Decoupling of Airborne Dynamic Bending Deformation Angle and Its Application in the High-Accuracy Transfer Alignment Process

**DOI:** 10.3390/s19010214

**Published:** 2019-01-08

**Authors:** Ping Yang, Xiyuan Chen, Junwei Wang

**Affiliations:** Key Laboratory of Micro-Inertial Instrument and Advanced Navigation Technology, Ministry of Education, School of Instrument Science and Engineering, Southeast University, Nanjing 210096, China; 220173238@seu.edu.cn (P.Y.); 220173220@seu.edu.cn (J.W.)

**Keywords:** dynamic deformation coupling angle, transfer alignment, angular velocity matching, airborne DPOS

## Abstract

In the traditional airborne distributed position and orientation system (DPOS) transfer alignment process, the coupling angle between the dynamic deformation and body angular motion is not estimated or compensated, which causes the process to have low precision and long convergence time. To achieve high-precision transfer alignment, a decoupling method for the airborne dynamic deformation angle is proposed in this paper. The model of the coupling angle is established through mathematical derivation. Then, taking the coupling angle into consideration, angular velocity error and velocity error between the master INS and slave IMU are corrected. Based on this, a novel 27-state Kalman filter model is established. Simulation results demonstrate that, compared with the traditional transfer alignment model, the model proposed in this paper has faster convergence time and higher accuracy.

## 1. Introduction

Over the years, with the improvement of imaging resolution of aeronautical earth observation systems and the demand for high-precision position and attitude reference information of three-dimensional images, the Distributed Position and Orientation System (DPOS) has been proposed and widely studied to determine high-precision motion parameters and time information for each observed load [1]. Airborne DPOS consists of the global positioning system, master inertial navigation system (INS) and several slave inertial measurement units (IMUs) [2]. The master INS is installed on the belly or in the cabin of the plane. The slave IMU, which consists of three orthogonal accelerometers and gyros, is in turn mounted near the mapping sensor to measure the slave nodes’ motion parameters [3,4,5,6]. The information measured by the slave IMU is transmitted to the master INS, then the attitude error and velocity error of the slave IMU is estimated by combining the high-precision navigation information of the master INS, which can be used to correct the initial state of the slave IMU; this process can be referred to as transfer alignment [7,8,9,10].

For high-performance integrated airborne earth observation systems equipped with multiple observation loads, each observation load is installed at different positions on the aircraft. At the same time, the aircraft is affected by loads and turbulence during flight, and its structure is dynamically deformed. This deformation introduces errors such as attitude, velocity, and angular velocity, which can decrease the accuracy of the transfer alignment between the master INS and slave IMU [11,12]. The traditional transfer alignment error model regards the aircraft wing as a rigid body, neglecting the dynamic deformation between the master INS and the slave IMUs; therefore, it is difficult to achieve high-precision measurement [13].

The model of the dynamic deformation of the aircraft wing can be simulated by the linear function of the specific force of the aircraft. The coefficient matrix of the linear function is closely related to the aircraft’s load, fuel quantity, flight speed and altitude [14]. However, the calculation of the model coefficient matrix is complex, and needs to be continuously updated according to the flight structure and flight state, making the model not compatible. In [15], the second-order Gauss–Markov is used to simulate the dynamic bending deformation angle, and the relevant bending deformation angle and angular rate are used as the state vectors of the Kalman filter, but the dynamic lever-arm is still regarded as constant during the velocity matching process. In [16], the lever arm and attitude error between the master INS and slave IMU under dynamic deformation are modeled and applied to the transfer alignment process, but the dynamic deformation is regarded as uncorrelated with the body motion. Browne [17] and Mochalov [18] have mentioned that the accuracy of the transfer alignment and the convergence time have a strong correlation with the motion of the body and the dynamic deformation. W. Wu analyzed this kind of coupled motion [19], and verified that there exists a relationship in amplitude and phase between the dynamic deformation and the motion of the body, but the results of the analysis cannot be directly applied to the transfer alignment process. In [20], a detailed mathematical analysis of the velocity relationship between the master INS and slave IMUs under dynamic deformation was carried out. The coupling relationship between the body motion and the dynamic deformation is also considered in the analysis. However, for airborne DPOS transfer alignment, the attitude and angular velocity matching methods are proved to be more suitable than the velocity matching method, because rapid maneuver will cause aircraft wing deformation, which will increase the estimation error of the lever arm, further decreasing the accuracy of velocity matching alignment [21,22]. 

This inspired our current study to investigate the coupling angle, and to apply it to compensate the angular velocity error for high precision transfer alignment. This paper is organized as follows. Section 2 establishes the model of the coupling angle. In Section 3, the angular velocity error and velocity error between the master INS and slave IMU are corrected by the coupling angle, through “attitude + velocity + angular velocity” matching method, the navigation errors of the slave IMUs are estimated in a 27-state Kalman filter which includes the coupling angle. Then the simulation experiments are performed and the results, which show that the coupling angle can improve the accuracy of transfer alignment, are presented in Section 4. The paper ends with the conclusion.

## 2. Coupled Error Angle Model of Dynamic Bending Deformation

**Figure 1:**
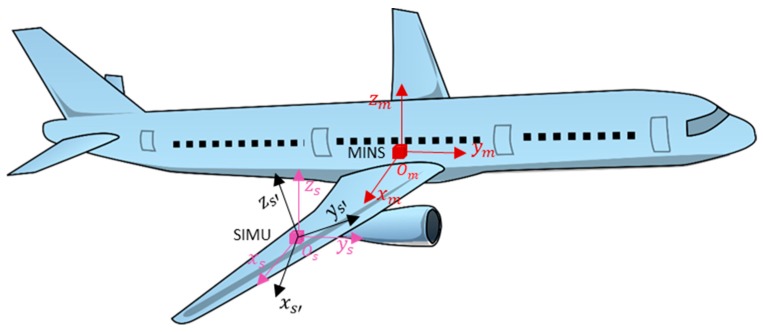
Sensor location.

As shown in Figure 1, the master INS is installed in the cabin or on the belly of the plane, while the slave IMUs are placed near the mapping sensors which are under the aircraft wing. In such a structure, there exist two motions between master INS and slave IMUs. One is rigid body motion, which represents movements whereby the aircraft’s structure is assumed to be rigid, meaning that relative motion does not exist between any two arbitrary positions on the aircraft wing in the body frame. The other is elastic body motion, composed of flexures and vibrations where there is relative motion between the points on the aircraft. Flexure represents the high-amplitude and low-frequency motion induced by the dynamic deformation of the aircraft while vibration defines the low-amplitude and high-frequency motion caused by forces from the change of the environment. Vibration is substituted by white noise in this paper [14].

### 2.1. Coordinate System Description

Before deriving the mathematical analysis, we define several coordinate systems commonly used in this paper. n denotes the navigation frame, which is the East-North-Up (E-N-U) geographic frame. e denotes the earth-centered earth-fixed frame. i denotes the earth-centered inertial frame. m denotes the body frame of the master INS. s denotes the ideal body frame of the slave IMU. s′ denotes the actual body frame of the slave IMU. The x-y-z-axis in the body frame of master and slave IMU points to East-North-Up.

### 2.2. Model of Coupling Angle 

The errorless angular velocity of the slave IMU can be represented by Equation (1):

(1) $$\omega =\omega_{stat}+\omega_{dyn},$$

with:

(2) $$\omega_{dyn}=\omega_{dyn}^{flex}+\omega_{dyn}^{\mathrm{vib}},$$

where:

ω is the errorless angular velocity of the slave IMU,
$\omega_{stat}$ is the static or invariant component of angular velocity, which is generated under rigid body motion,
$\omega_{dyn}$ is the dynamic component of angular velocity, which is generated under elastic body motion,
$\omega_{dyn}^{flex}$ is the component of $\omega_{dyn}$ due to flexure motion,
$\omega_{dyn}^{\mathrm{vib}}$ is the component of $\omega_{dyn}$ vibration motion, which is assumed to be white noise.

The flexural angle between the master INS and slave IMU is simulated by the second-order Gauss–Markov process [16]:

(3) $${\ddot{\theta }}_{i}=-2\beta_{i}{\dot{\theta }}_{i}-\beta_{i}^{2}\theta_{i}+\varpi_{i}\;(i=x,y,z),$$

where $\theta ={\left[\begin{array}{ccc} \theta_{x} & \theta_{y} & \theta_{z} \end{array}\right]}^{T}$ is flexural angle; the covariance of flexural angle θ is $\sigma_{i}^{2}$, $\beta_{i}=2.146/\tau_{i}$ and $\tau_{i}$ is the correlation time; $\varpi_{i}$ is the Gaussian white noise with covariance as follow:

(4) $$Q_{i}=4\beta_{i}^{3}\sigma_{i}^{2}.$$

Then flexural angular velocity is shown in Equation (5):

(5) $$\omega_{dyn}^{flex}=\dot{\theta }=\omega_{\theta }.$$

The theoretical error angle between the master INS and slave IMU can be formulated as:

(6) $$\varphi =\rho_{0}+\theta ,$$

where $\rho_{0}$ and θ represent the rigid misalignment angle $C_{m}^{s}(\varphi )$ and flexural angle between the master INS and slave IMU, respectively.

The ideal angular velocity of the s-frame with respect to the i-frame can be represented as follows:

(7) $$\omega_{is}^{s}=C_{m}^{s}(\varphi )\omega_{im}^{m},$$

where $C_{m}^{s}(\varphi )$ is the theoretical direction cosine matrix from m frame to s-frame, and $\omega_{im}^{m}$ means the angular velocity between m frame and the i-frame.

However, there is a coupling angle Δϕ and flexural angular velocity $\omega_{\theta }$ that are due to the coupling influence of rigid and elastic body motion between the master INS and the slave IMUs. Therefore, the actual angular velocity of the s-frame with respect to the i-frame can be defined as:

(8) $$\omega_{is}^{s\prime }=\omega_{is}^{s}+\omega_{\theta },$$

where $\omega_{is}^{s}={\left[\begin{array}{ccc} \omega_{isx}^{s} & \omega_{isy}^{s} & \omega_{isz}^{s} \end{array}\right]}^{T}$, $\omega_{\theta }={\left[\begin{array}{ccc} \omega_{\theta x} & \omega_{\theta y} & \omega_{\theta z} \end{array}\right]}^{T}$, and we have:

(9) $$\omega_{is}^{s\prime }={\left[\begin{array}{ccc} \omega_{isx}^{s}+\omega_{\theta x} & \omega_{isy}^{s}+\omega_{\theta y} & \omega_{isz}^{s}+\omega_{\theta z} \end{array}\right]}^{T},$$

**Figure 2:**
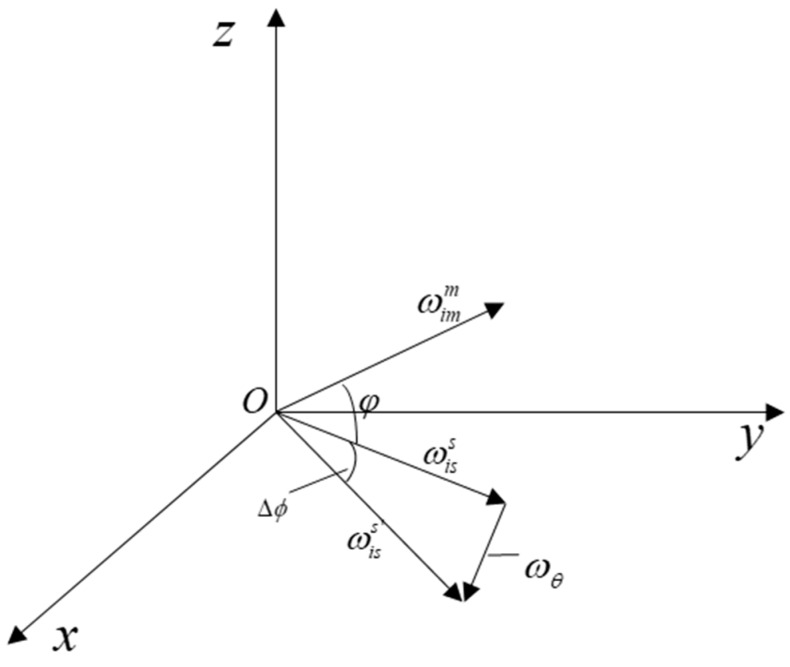
The relationship between $\omega_{im}^{m}$, $\omega_{is}^{s}$, $\omega_{\theta }$ and $\omega_{is}^{s\prime }$.

The relationship between $\omega_{im}^{m}$, $\omega_{is}^{s}$, $\omega_{\theta }$ and $\omega_{is}^{s\prime }$ is shown in Figure 2.

The coupling angle Δϕ represents the angle between $\omega_{is}^{s}$ and $\omega_{is}^{s\prime }$, it can be described as Equation (10):

(10) $$\Delta \phi ={\left[\begin{array}{ccc} \Delta \phi_{x} & \Delta \phi_{y} & \Delta \phi_{z} \end{array}\right]}^{T},$$

**Figure 3:**
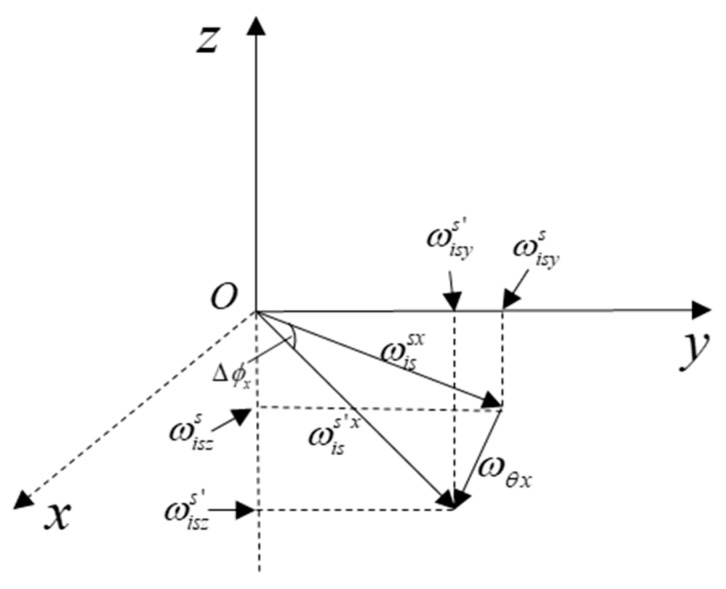
The x-axis coupling error angle.

Firstly, the coupling angle $\Delta \phi_{x}$ related to the flexural angular velocity $\omega_{\theta x}$ is shown in Figure 3.

From the above figure, and combining with Equation (9), we have:

(11) $$\Delta \phi_{x}=\arctan\frac{\omega_{isz}^{s}}{\omega_{isy}^{s}}-\arctan\frac{\omega_{isz}^{s}+\omega_{\theta z}}{\omega_{isy}^{s}+\omega_{\theta y}},$$

Correspondingly, by utilizing the aforementioned method, we can obtain:

(12) $$\{\begin{array}{c} \Delta \phi_{x}=\arctan\frac{\omega_{isz}^{s}}{\omega_{isy}^{s}}-\arctan\frac{\omega_{isz}^{s}+\omega_{\theta z}}{\omega_{isy}^{s}+\omega_{\theta y}}\\ \Delta \phi_{y}=\arctan\frac{\omega_{isx}^{s}}{\omega_{isz}^{s}}-\arctan\frac{\omega_{isx}^{s}+\omega_{\theta x}}{\omega_{isz}^{s}+\omega_{\theta z}}\\ \Delta \phi_{z}=\arctan\frac{\omega_{isy}^{s}}{\omega_{isx}^{s\prime }}-\arctan\frac{\omega_{isy}^{s}+\omega_{\theta y}}{\omega_{isx}^{s}+\omega_{\theta x}} \end{array}.$$

Then the trigonometric Taylor series expansion is applied to Equation (12), the coupling angle can be rewritten as Equation (13):

(13) $$\{\begin{array}{c} \Delta \phi_{x}=\frac{\omega_{isz}^{s}}{\omega_{isy}^{s}}-\frac{\omega_{isz}^{s}+\omega_{\theta z}}{\omega_{isy}^{s}+\omega_{\theta y}}\\ \Delta \phi_{y}=\frac{\omega_{isx}^{s}}{\omega_{isz}^{s}}-\frac{\omega_{isx}^{s}+\omega_{\theta x}}{\omega_{isz}^{s}+\omega_{\theta z}}\\ \Delta \phi_{z}=\frac{\omega_{isy}^{s}}{\omega_{isx}^{s}}-\frac{\omega_{isy}^{s}+\omega_{\theta y}}{\omega_{isx}^{s}+\omega_{\theta x}} \end{array}.$$

Furthermore, since $\omega_{\theta }$ is much smaller than $\omega_{is}^{s}$, Equation (13) can be simplified as follows:

(14) $$\Delta \phi =M\omega_{\theta }=M\dot{\theta },$$

where the coefficient matrix M can be described as:

(15) $$M=\left[\begin{array}{ccc} 0 & 0 & -\frac{1}{\omega_{isy}^{s}}\\ -\frac{1}{\omega_{isz}^{s}} & 0 & 0\\ 0 & -\frac{1}{\omega_{isx}^{s}} & 0 \end{array}\right].$$

## 3. Transfer Alignment Model 

### 3.1. Model of Angular Velocity Error and Compensation

**Figure 4:**
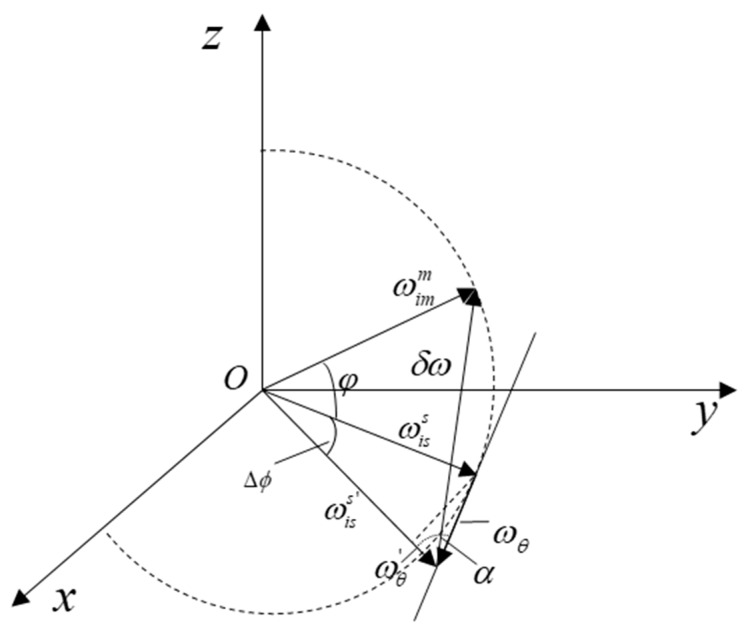
The angular velocity error between $\omega_{im}^{m}$ and $\omega_{is}^{s\prime }$.

The angular velocity error between $\omega_{im}^{m}$ and $\omega_{is}^{s\prime }$ is shown in Figure 4. From the figure, the angular velocity error δω can be calculated by Equation (16):

(16) $$\delta \omega =\omega_{is}^{s\prime }-\omega_{im}^{m}=C_{m}^{s\prime }(\varphi +\Delta \phi )\omega_{im}^{m}+\omega_{\theta }^{\prime }-\omega_{im}^{m},$$

where $C_{m}^{s\prime }(\varphi +\Delta \phi )$ is the actual direction cosine matrix that transforms a vector from m-frame projection form to the s′-frame projection form, it can be approximately expressed:

(17) $$C_{m}^{s\prime }(\varphi +\Delta \phi )\approx \left[\begin{array}{ccc} 1 & {\left(\varphi +\Delta \phi \right)}_{z} & -{\left(\varphi +\Delta \phi \right)}_{y}\\ -{\left(\varphi +\Delta \phi \right)}_{z} & 1 & {\left(\varphi +\Delta \phi \right)}_{x}\\ {\left(\varphi +\Delta \phi \right)}_{y} & -{\left(\varphi +\Delta \phi \right)}_{x} & 1 \end{array}\right]=I-[(\varphi +\Delta \phi )\times ],$$

where (φ+Δϕ) is the actual error angle between the master INS and slave IMU. Through combining Equations (16) and (17), the angular velocity error δω can be achieved as follows:

(18) $$\delta \omega =\omega_{\theta }^{\prime }-[(\varphi +\Delta \phi )\times ]\omega_{im}^{m},$$

where $\omega_{\theta }^{\prime }$ is the projection of $\omega_{\theta }$ onto $\omega_{is}^{s\prime }$, and its direction is in accordance with $\omega_{is}^{s\prime }$. Hence $\omega_{\theta }^{\prime }$ can be formulated as:

(19) $$\omega_{\theta }^{\prime }=A(\omega_{\theta })T(\alpha )u_{is}^{s\prime },$$

since the coupling angle Δϕ is a small angle, its direction can be approximated along the tangential direction, so the angle between the vectors $\omega_{\theta }$ and $\omega_{is}^{s\prime }$ can be represented by Equation (20):

(20) $$\alpha =\frac{\pi }{2}U-\Delta \phi ,$$

where $U={\left[\begin{array}{ccc} 1 & 1 & 1 \end{array}\right]}^{T}$, $A(\omega_{\theta })$ is the magnitude matrix, T(α) is the direction cosine matrix, and $u_{is}^{s\prime }$ is the unit direction vector. These can be expressed, respectively, as:

$$A(\omega_{\theta })=\left[\begin{array}{ccc} \left|\omega_{\theta_{x}}\right| & 0 & 0\\ 0 & \left|\omega_{\theta_{y}}\right| & 0\\ 0 & 0 & \left|\omega_{\theta_{z}}\right| \end{array}\right],\;T(\alpha )=I-\left[(\frac{\pi }{2}U-\Delta \phi )\times \right],\;u_{is}^{s\prime }=\frac{\omega_{is}^{s\prime }}{\left|\omega_{is}^{s\prime }\right|},$$

where $(\frac{\pi }{2}U-\Delta \phi )\times$ is the skew-symmetric matrix of $\left(\frac{\pi }{2}U-\Delta \phi \right)$.

Substituting Equation (19) into Equation (18) yields:

(21) $$\delta \omega =(\omega_{im}^{m}\times )(\rho +\theta )+(\omega_{im}^{m}\times +\frac{A(\omega_{\theta })}{\left|\omega_{is}^{s\prime }\right|}\omega_{is}^{s\prime }\times )\Delta \phi +A(\omega_{\theta })\frac{\omega_{is}^{s\prime }}{\left|\omega_{is}^{s\prime }\right|}-\frac{\pi }{2}A(\omega_{\theta })(U\times )\frac{\omega_{is}^{s\prime }}{\left|\omega_{is}^{s\prime }\right|}.$$

### 3.2. Model of Velocity Error and Compensation

**Figure 5:**
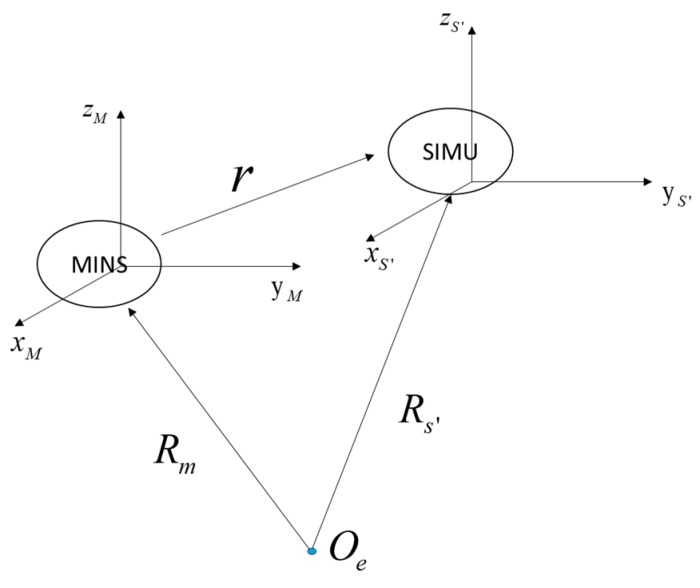
The relative positional relationship between $R_{m}$, $R_{m}$ and r.

The position relationship between the master INS and slave IMU is shown in Figure 5. The position vector of the master INS and the slave IMU in the e-frame can be described as $R_{m}$ and $R_{s\prime }$, and the lever-arm between the master INS and the slave IMU can be expressed as r [16]. Therefore, we have:

(22) $$R_{s\prime }=R_{m}+r,$$

Under the i-frame, Equation (22) can be expressed as:

(23) $$R_{s\prime }^{i}=R_{m}^{i}+C_{m}^{i}r^{m},$$

where $C_{m}^{i}$ is the direction cosine matrix from the m-frame to i-frame; by differentiating on both sides of Equation (23), we have:

(24) $${\ddot{R}}_{s\prime }^{i}={\ddot{R}}_{m}^{i}+C_{m}^{i}(\omega_{im}^{m}\times )(\omega_{im}^{m}\times )r^{m}+C_{m}^{i}({\dot{\omega }}_{im}^{m}\times )r^{m}+C_{m}^{i}(\omega_{im}^{m}\times ){\dot{r}}^{m}+C_{m}^{i}(\omega_{im}^{m}\times ){\dot{r}}^{m}+C_{m}^{i}{\ddot{r}}^{m}.$$

According to Newton’s second law of motion, we have:

(25) $$\begin{array}{c} {\ddot{R}}_{s\prime }^{i}=f_{s\prime }^{i}+g_{s\prime }^{i}+\omega_{ie}^{i}\times (\omega_{ie}^{i}\times R_{s\prime }^{i})\\ {\ddot{R}}_{m}^{i}=f_{m}^{i}+g_{m}^{i}+\omega_{ie}^{i}\times (\omega_{ie}^{i}\times R_{m}^{i}) \end{array},$$

where $f_{s\prime }^{i}$ and $f_{m}^{i}$ are projections of specific force in earth-centered inertial frame, $\omega_{ie}^{i}\times (\omega_{ie}^{i}\times R_{s\prime }^{i})$ and $\omega_{ie}^{i}\times (\omega_{ie}^{i}\times R_{m}^{i})$ are the Coriolis accelerations; $g_{s\prime }^{i}$ and $g_{m}^{i}$ are the gravity accelerations. 

Assuming that $g_{s\prime }^{i}=g_{m}^{i}$, substituting Equation (25) into Equation (24) yields:

(26) $$f_{s\prime }^{i}=f_{m}^{i}+C_{m}^{i}(\omega_{im}^{m}\times )(\omega_{im}^{m}\times )r^{m}+C_{m}^{i}({\dot{\omega }}_{im}^{m}\times )r^{m}+C_{m}^{i}(\omega_{im}^{m}\times ){\dot{r}}^{m}+C_{m}^{i}(\omega_{im}^{m}\times ){\dot{r}}^{m}+C_{m}^{i}{\ddot{r}}^{m},$$

multiply $C_{i}^{m}$ on both sides of Equation (26) and rearrange as follows:

(27) $$C_{s\prime }^{m}f_{s\prime }^{s\prime }=f_{m}^{m}+(\omega_{im}^{m}\times )(\omega_{im}^{m}\times )r^{m}+({\dot{\omega }}_{im}^{m}\times )r^{m}+(\omega_{im}^{m}\times ){\dot{r}}^{m}+(\omega_{im}^{m}\times ){\dot{r}}^{m}+{\ddot{r}}^{m},$$

where $C_{s\prime }^{m}$ represents the direction cosine matrix from s′-frame to m-frame.

The velocity differential equation of the master INS and the slave IMU can be obtained:

(28) $$\begin{array}{c} {\dot{v}}_{m}^{n}=C_{m}^{n}f_{m}^{m}-(2\omega_{ie}^{n}+\omega_{en}^{n})\times v_{m}^{n}+g^{n}\\ {\dot{v}}_{s\prime }^{n}=C_{m}^{n}C_{s\prime }^{m}(f_{s\prime }^{s\prime }+\nabla^{s\prime })-(2\omega_{ie}^{n}+\omega_{en}^{n})\times v_{s\prime }^{n}+g^{s\prime } \end{array},$$

where $v_{m}^{n}$ and $v_{s\prime }^{n}$ represent the velocity of the master INS and the velocity of the slave IMU in navigation frame, respectively. $\nabla^{s\prime }$ is the accelerometer bias of the slave IMU. The direction cosine matrix from s′-frame to s-frame is given as:

(29) $$C_{s\prime }^{s}=I-(\phi \times ),$$

where ϕ is the equivalent rotation vector between s′-frame and s-frame. The velocity error δv is defined by Equation (30):

(30) $$\delta v=v_{s\prime }^{n}-v_{m}^{n}.$$

Differentiate the two sides of Equation (30), and substituting Equations (27) and (28) into Equation (30) yields:

(31) $$\delta \dot{v}=-(2\omega_{ie}^{n}+\omega_{en}^{n})\times \delta v+[(\omega_{im}^{n}\times )(\omega_{im}^{n}\times )+({\dot{\omega }}_{im}^{n}\times )]r^{n}+2(\omega_{im}^{n}\times ){\dot{r}}^{n}+{\ddot{r}}^{n}+C_{s\prime }^{n}\nabla^{s\prime }+(f_{s}^{n}\times )\phi ,$$

then from [20], we can obtain:

(32) $$\begin{array}{c} r=r_{0}+\delta r\\ \delta r=R_{0}(\theta +\Delta \phi ) \end{array},$$

where $r_{0}={\left[\begin{array}{ccc} x_{0} & y_{0} & z_{0} \end{array}\right]}^{T}$ and $R_{0}=\left[\begin{array}{ccc} 0 & z_{0} & 0\\ 0 & 0 & x_{0}\\ y_{0} & 0 & 0 \end{array}\right]$, then differentiate both sides of Equation (32), we have:

(33) $$\begin{array}{c} \dot{r}=\delta \dot{r}=R_{0}(\dot{\theta }+\Delta \dot{\phi })\\ \ddot{r}=R_{0}(\ddot{\theta }+\Delta \ddot{\phi })=R_{0}B_{1}\dot{\theta }+R_{0}B_{2}\theta +R_{0}\Delta \ddot{\phi } \end{array},$$

from Equation (3), we can obtain:

(34) $$B_{1}=\left[\begin{array}{ccc} 0 & -2\beta_{y} & 0\\ 0 & 0 & -2\beta_{z}\\ -2\beta_{x} & 0 & 0 \end{array}\right]{\;B}_{2}=\left[\begin{array}{ccc} 0 & -\beta_{y}^{2} & 0\\ 0 & 0 & -\beta_{z}^{2}\\ -\beta_{x}^{2} & 0 & 0 \end{array}\right].$$

Substituting Equations (33) and (34) into Equation (31) yields:

(35) $$\begin{array}{ll} \delta \dot{v} & =-(2\omega_{ie}^{n}+\omega_{en}^{n})\times \delta v+R_{0}B_{2}\theta +(2(\omega_{im}^{n}\times )R_{0}+R_{0}B_{1})\dot{\theta }+C_{s\prime }^{n}\nabla^{s}+R_{0}\Delta \ddot{\phi }\\ & +[(\omega_{im}^{n}\times )(\omega_{im}^{n}\times )+({\dot{\omega }}_{im}^{n}\times )]\delta r+(f_{s}^{n}\times )\phi +[(\omega_{im}^{n}\times )(\omega_{im}^{n}\times )+({\dot{\omega }}_{im}^{n}\times )]r_{0} \end{array}.$$

### 3.3. State Equation

The state equation of the system can be formulated as

(36) $$\dot{x}=Fx+Gw,$$

where the state vector x can be expressed as follows:

(37) $$x={\left[\begin{array}{ccccccccc} \phi & \delta v & \varepsilon & \nabla & \rho_{0} & \theta & \dot{\theta } & \delta r & \Delta \phi \end{array}\right]}^{T},$$

The differential equation of the attitude error between s′-frame and s-frame is given by Equation (36):

(38) $$\dot{\phi }=-\omega_{in}^{n}\times \phi -C_{s\prime }^{n}\varepsilon^{s\prime },$$

where $C_{s\prime }^{n}$ is the direction cosine matrix from s′-frame to n-frame.

The differential equation of the coupling angle is formulated by Equation (39):

(39) $$\begin{array}{c} \Delta \dot{\phi }=M{\dot{\omega }}_{\theta }=M\ddot{\theta }\\ \Delta \ddot{\phi }=M\dddot{\theta }=M(B_{1}\ddot{\theta }+B_{2}\dot{\theta })=M(B_{1}^{2}\dot{\theta }+(B_{1}B_{2}+B_{2})\theta ) \end{array}.$$

Integrating Equations (3), (33), (35), (38) and (39), we obtain the expression of the state matrix as follows:

(40) $$F=[\begin{array}{ccccccccc} \left(-\omega_{in}^{n}\times \right) & 0_{3\times 3} & -C_{s'}^{n} & 0_{3\times 3} & 0_{3\times 3} & 0_{3\times 3} & 0_{3\times 3} & 0_{3\times 3} & 0_{3\times 3}\\ \left(f_{s}^{n}\times \right) & F_{22} & 0_{3\times 3} & C_{s'}^{n} & 0_{3\times 3} & F_{26} & F_{27} & F_{28} & 0_{3\times 3}\\ 0_{3\times 3} & 0_{3\times 3} & 0_{3\times 3} & 0_{3\times 3} & 0_{3\times 3} & 0_{3\times 3} & 0_{3\times 3} & 0_{3\times 3} & 0_{3\times 3}\\ 0_{3\times 3} & 0_{3\times 3} & 0_{3\times 3} & 0_{3\times 3} & 0_{3\times 3} & 0_{3\times 3} & 0_{3\times 3} & 0_{3\times 3} & 0_{3\times 3}\\ 0_{3\times 3} & 0_{3\times 3} & 0_{3\times 3} & 0_{3\times 3} & 0_{3\times 3} & 0_{3\times 3} & 0_{3\times 3} & 0_{3\times 3} & 0_{3\times 3}\\ 0_{3\times 3} & 0_{3\times 3} & 0_{3\times 3} & 0_{3\times 3} & 0_{3\times 3} & 0_{3\times 3} & I_{3\times 3} & 0_{3\times 3} & 0_{3\times 3}\\ 0_{3\times 3} & 0_{3\times 3} & 0_{3\times 3} & 0_{3\times 3} & 0_{3\times 3} & B_{1} & B_{2} & 0_{3\times 3} & 0_{3\times 3}\\ 0_{3\times 3} & 0_{3\times 3} & 0_{3\times 3} & 0_{3\times 3} & 0_{3\times 3} & F_{86} & F_{87} & 0_{3\times 3} & 0_{3\times 3}\\ 0_{3\times 3} & 0_{3\times 3} & C_{s^{\prime }}^{n} & 0_{3\times 3} & 0_{3\times 3} & F_{96} & F_{97} & 0_{3\times 3} & 0_{3\times 3} \end{array}]$$

where $F_{22}=-[(2\omega_{ie}^{n}+\omega_{en}^{n})\times ]$, $F_{26}=R_{0}B_{2}+R_{0}M(B_{1}B_{2}+B_{2})$, $F_{27}=(2(\omega_{im}^{n}\times )R_{0}+R_{0}B_{1})+R_{0}MB_{1}^{2}$ $F_{28}=[(\omega_{im}^{n}\times )(\omega_{im}^{n}\times )+({\dot{\omega }}_{im}^{n}\times )]$, $F_{86}=R_{0}MB_{2}$, $\;F_{87}=R_{0}+R_{0}MB_{1}$, $F_{96}=MB_{2}$ and $F_{97}=MB_{1}$.

### 3.4. Measurement Equation

In the process of transfer alignment of the aeronautical earth observation system, the ideal flight status is that the speed of the body is constant while the attitude angle changes with the flight state. Hence, the “attitude + velocity + angular velocity” matching model is very suitable. On the one hand, for airborne DPOS transfer alignment, “attitude + velocity + angular velocity” matching methods are proved to be more suitable than “attitude + velocity” matching. On the other hand, using this matching method, the alignment accuracy is improved, and the alignment time is shortened. 

The system state equation can be described as:

(41) y=Hx+ν,

The measurement vector y can be selected as follows:

(42) $$y={\left[\begin{array}{ccccccccc} \delta \phi_{E} & \delta \phi_{N} & \delta \phi_{U} & \delta \nu_{E} & \delta \nu_{N} & \delta \nu_{U} & \delta \omega_{E} & \delta \omega_{N} & \delta \omega_{U} \end{array}\right]}^{T}.$$

From Equation (21), we have:

(43) $$\delta \omega =\left[\begin{array}{ccccccccc} 0_{3\times 3} & 0_{3\times 3} & 0_{3\times 3} & 0_{3\times 3} & \left(\omega_{im}^{m}\times \right) & \left(\omega_{im}^{m}\times \right) & I_{3\times 3} & 0_{3\times 3} & H_{4} \end{array}\right]x,$$

where $H_{4}=(\omega_{im}^{m}\times +\frac{A(\omega_{\theta })}{\left|\omega_{is}^{s}\right|}\omega_{is}^{s}\times )$.

As for δϕ we have:

(44) $$\delta \phi =\left[\begin{array}{ccccccccc} H_{1} & 0_{3\times 3} & H_{2} & H_{3} & 0_{3\times 3} & 0_{3\times 3} & 0_{3\times 3} & 0_{3\times 3} & 0_{3\times 3} \end{array}\right]x,$$

where $H_{1}$, $H_{2}$ and $H_{3}$ can be found in [15].

Integrating Equations (43) and (44), we get the corresponding measurement matrix:

(45) $$H=\left[\begin{array}{ccccccccc} H_{1} & 0_{3\times 3} & H_{2} & H_{3} & 0_{3\times 3} & 0_{3\times 3} & 0_{3\times 3} & 0_{3\times 3} & 0_{3\times 3}\\ 0_{3\times 3} & I_{3\times 3} & 0_{3\times 3} & 0_{3\times 3} & 0_{3\times 3} & 0_{3\times 3} & 0_{3\times 3} & 0_{3\times 3} & 0_{3\times 3}\\ 0_{3\times 3} & 0_{3\times 3} & 0_{3\times 3} & 0_{3\times 3} & \left(\omega_{im}^{m}\times \right) & \left(\omega_{im}^{m}\times \right) & I_{3\times 3} & 0_{3\times 3} & H_{4} \end{array}\right].$$

### 3.5. Filter Selection

Since the Kalman filter provides the optimal estimate of the states of a stochastic dynamical system if the system is linear, the measurements are also linear functions of states and the errors in system modeling and the measurements are Gaussian white noise. At the same time, from the mod-el which was established in this paper, lever-arm is the linear function of bending deformation angle, and the coupling angle is the linear function of bending deformation angle rate. Finally, the linear estimator chosen for the transfer alignment of airborne DPOS was the Kalman filter. 

## 4. Simulation and Results

**Figure 6:**
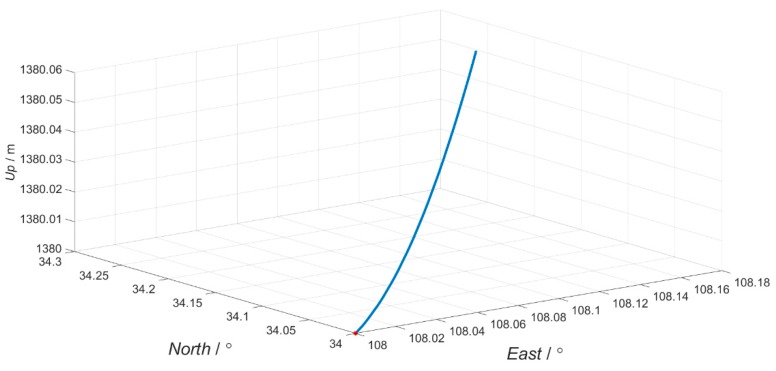
The flight trajectory.

**Figure 7:**
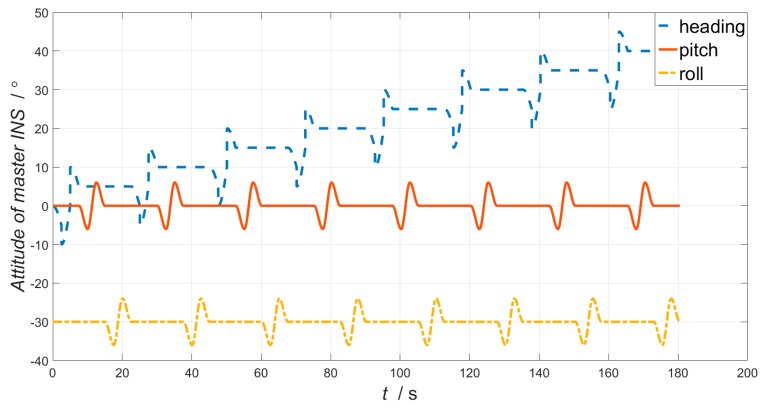
The attitude of master INS.

The basic principle of the high-resolution mapping system requires the aerial sensors to move at constant speed along a linear trajectory. However, in practical flight, due to the influence of atmospheric turbulence, equipment performance and other factors, the aircraft has interference effects in with respect to flight direction and lateral direction, resulting in changes in the attitude angle. Therefore, the simulation flight trajectory is approximate to a straight line, as shown in Figure 6. The initial longitude and latitude we choose for simulation are 108°, 34°. The simulation time is 180 s. The simulated position, velocity, attitude, and raw measurement data of the accelerometer and gyroscope are used as the navigation parameters of the master INS. Attitude angle for master INS is shown in Figure 7.

The master INS data, the flexural angle θ and the rigid misalignment angle $\rho_{0}$ are combined as the output of the simulated IMU data of slave IMU. $\rho_{0}=\left[\begin{array}{ccc} 10^{{}^{\circ}} & 10^{{}^{\circ}} & 5^{{}^{\circ}} \end{array}\right]$ is the rigid misalignment angle. $\tau =\left[\begin{array}{ccc} 60 & 60 & 60 \end{array}\right]$ represents the correlation time of the second-order Gauss–Markov process. The variance of flexural angle is set as $\sigma =\left[\begin{array}{ccc} 0.1^{{}^{\circ}} & 0.1^{{}^{\circ}} & 0.1^{{}^{\circ}} \end{array}\right]$. The initial lever-arm is set as $R_{0}=\left[\begin{array}{ccc} 3.6m & 0.15m & 0.25m \end{array}\right]$. The sensors’ specifications of master INS and slave IMUs are shown in Table 1.

**Figure 8:**
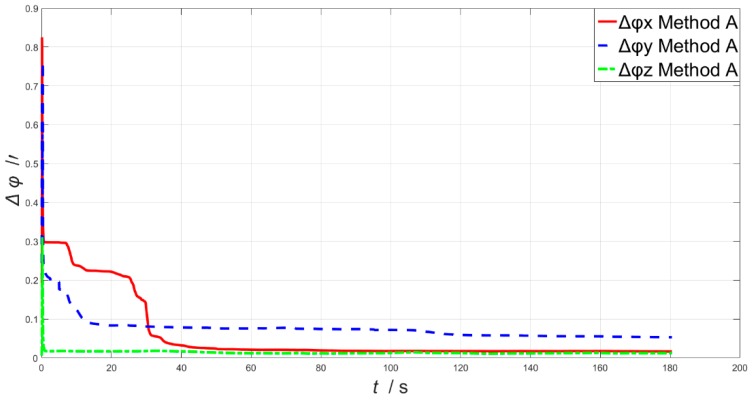
The estimation errors of the coupling angles.

A model which takes the coupling angle into consideration as proposed in this paper (Method A), as well as a model which neglects the coupling angle (Method B) are performed for comparison. The estimation errors of the coupling angles are presented in Figure 8. As is shown in Figure 8, estimation errors of the coupling angles converge fast, and the root-mean-square errors (RMSE) of the *x-axis*, *y-axis* and *z-axis* are 0.101349’, 0.083983’ and 0.016011’, respectively.

**Figure 9:**
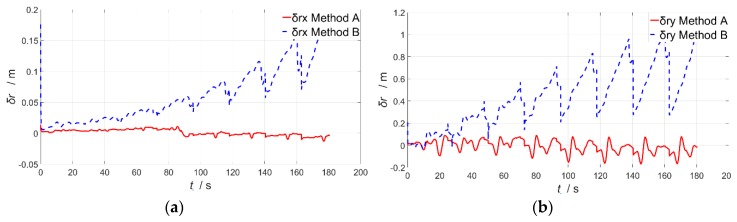

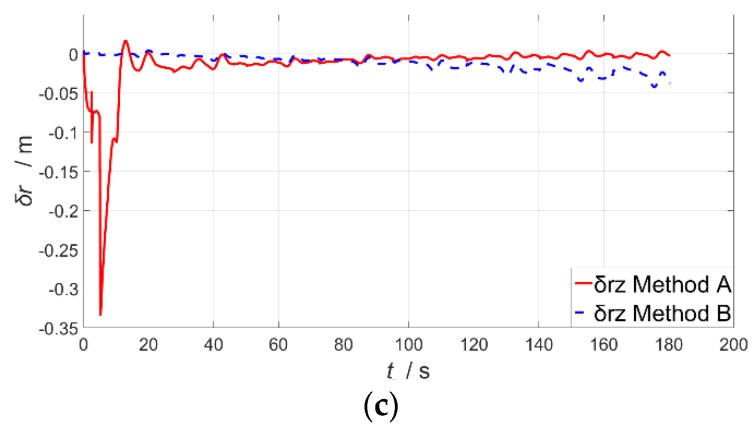
The estimation errors of the coupling angles. (**a**) The x-axis dynamic lever arm error; (**b**) The y-axis dynamic lever arm error; (**c**) The z-axis dynamic lever arm error.

The estimation errors’ variations of the dynamic lever-arm are shown in Figure 9a–c. The RMSE of the dynamic lever arm is shown in Table 2. Figure 9 shows that the estimation errors of the flexural lever arm can be estimated properly by Method A, while it is not stable when using Method B. Meanwhile, from Table 2, we can draw the conclusion that the errors of the dynamic lever arm are much smaller by Method A proposed in this paper than Method B.

**Figure 10:**
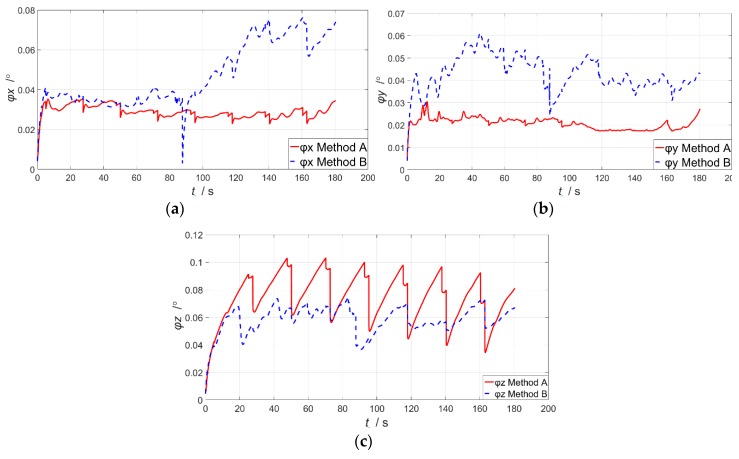
The estimation errors of attitude. (**a**) The x-axis attitude error; (**b**) The y-axis attitude error; (**c**) The z-axis attitude error.

Figure 10a–c shows the estimation errors of attitude variations, and the RMSE is presented in Table 3. It is clear that Method A converges more steadily and faster. Hence, with better accuracy and shorter convergence time, Method A is demonstrated to be more capable for the practical information measurement process in DPOS. From Table 3, we conclude that Method A, which is proposed in this paper, has higher precision than Method B on the estimation of attitude. The RMSE of method A is reduced to 92.9% of that of method B on average. 

## 5. Discussion

This paper has proposed a method which can be used to estimate the coupling angle caused by the coupling influence of dynamic flexure with aircraft motion and compensate velocity error, as well as angular velocity error, to improve the estimation accuracy while using an angular velocity matching method in the transfer alignment process. By taking the correlation between the dynamic deformation and body motion into consideration, we established a model based on angular velocity, velocity and coupling angle. Then, this model was used to correct the angular velocity error and dynamic lever-arm. An “attitude + velocity + angular velocity” matching method was applied to estimate the coupling angle and attitude error. Simulation results demonstrated that, compared to the traditional method, which assumes the dynamic deformation and body motion to be uncorrelated, the model developed in this paper can effectively estimate the flexural lever arm and the coupling angle, thus compensating the angular velocity and velocity error.

In practice, aircraft experience different circumstances, such as strong wind, turbulence and other factors, and the deformation of the aircraft wing is great. Thus, the nonlinearity of the airborne DPOS increases. In addition, the measurement results of the system are utilized in the off-line states. Thence the change of the aircraft wing’s parameters introduced by environment changes can influence the accuracy of the results. Further studies should be performed with regard to a nonlinear filter such as EKF and a nonlinear model to compensate the angular velocity error and lever-arm in order to realize the higher-resolution aerial mapping.

## Figures and Tables

**Table 1:** The sensors’ specifications of master INS and slave IMUs.

		Master INS	Slave IMU
gyroscope	random drift	$0.005^{{}^{\circ}}/h$	$0.1^{{}^{\circ}}/h$
	white noise	$0.002^{{}^{\circ}}/\sqrt{h}$	$0.02^{{}^{\circ}}/\sqrt{h}$
accelerometer	random drift	10μg	100μg
	white noise	$0.05\mu g\sqrt{h}$	$0.2\mu g\sqrt{h}$

**Table 2:** The RMSE of the dynamic lever arm.

Estimation Error	Method A	Method B
$\delta r_{x}$(m)	0.004998	0.074664
$\delta r_{y}$(m)	0.056484	0.499977
$\delta r_{z}$(m)	0.037366	0.015182

**Table 3:** The RMSE of attitude error.

Estimation Error	Method A	Method B
$\phi_{x}$(°)	0.021147	0.043164
$\phi_{y}$(°)	0.019393	0.061425
$\phi_{z}$(°)	0.078595	0.045374
